# Towards an improved definition of periprocedural myocardial infarction: The role of high‐sensitivity cardiac troponins

**DOI:** 10.1111/jocs.16107

**Published:** 2021-10-24

**Authors:** Samuel Heuts, Iwan C. C. van der Horst, Alma Mingels

**Affiliations:** ^1^ Department of Cardiothoracic Surgery Maastricht University Medical Center+ Maastricht The Netherland; ^2^ Department of Intensive Care Medicine Maastricht University Medical Center+ Maastricht The Netherland; ^3^ Cardiovascular Research Institute Maastricht (CARIM) Maastricht University Maastricht The Netherland; ^4^ Central Diagnostic Laboratory Maastricht University Medical Center+ Maastricht The Netherland

**Keywords:** cardiac troponin, high‐sensitivity cardiac troponin T, periprocedural myocardial infarction

## Abstract

In the past few years, many have disputed the optimal biomarker for confirming or ruling out a diagnosis of periprocedural myocardial infarction (PMI) and the optimal cut‐off concentrations to apply. In this issue of the *Journal of Cardiac Surgery*, Niclauss et al. performed a retrospective analysis of CK‐MB and high‐sensitivity cardiac troponin T (hs‐cTnT) dynamics and peak concentrations following different cardiac surgical interventions in 400 patients during a 2‐year period in a single center. The authors found that CK‐MB and hs‐cTnT predict PMI with a comparable diagnostic accuracy and discriminatory power >95%. They also attempted to propose an improved, more sensitive threshold of hs‐cTnT for PMI. Their findings could have implications for clinical practice, but more research is warranted to identify more appropriate cut‐offs. This could include hs‐cTnT release pattern, slope steepness, and changes. Ultimately, this could results in patient‐specific model, able to predict expected and abnormal ranges of hs‐cTnT release, enabling an improved and timely diagnosis of PMI.

1

In the past few years, many have disputed the optimal biomarker for confirming or ruling out a diagnosis of periprocedural myocardial infarction (PMI) and the optimal cut‐off concentrations to apply.^1^ Moreover, recent controversies emphasized the need for a uniform definition following the 5‐year results of the *Evaluation of XIENCE versus Coronary Artery Bypass Surgery for Effectiveness of Left Main Revascularization* (EXCEL)‐trial.^2^, ^3^ These controversies arose from the apparent selective use of conflicting definitions of myocardial infarction, namely the Universal Definition (UDMI‐4)^4^ and the definition proposed by the Society for Cardiovascular Angiography and Interventions (SCAI).^5^ Two topics of utmost interest in these “rivaling” definitions are (1) the use of *isolated* biomarker concentration elevations^5^ versus a biomarker rise accompanied by *additional evidence* (electrocardiographic, echocardiographic, or angiographic),^4^ and (2) the preferred use of either the MB‐isoenzyme of creatine kinase (CK‐MB)^5^ or cardiac troponins.^4^, ^6^

In this issue of *the Journal of Cardiac Surgery*, Niclauss et al.^7^ performed a retrospective analysis of CK‐MB and high‐sensitivity cardiac troponin T (hs‐cTnT) dynamics and peak concentrations following different cardiac surgical interventions in 400 patients during a 2‐year period in a single center. The majority of patients (42%) underwent coronary artery bypass grafting (CABG; on‐pump in 71% of cases, off‐pump [OPCAB] in 29%), other procedures included isolated aortic valve replacement (AVR; 25%), combined CABG + AVR (13%), mitral valve surgery (14%), and a concomitant rate of 18% for septal myectomies. The median peak concentrations of these biomarkers were compared to the UDMI‐4 to evaluate their accuracy for diagnosis of PMI using a receiver operating characteristics analysis. Definite PMI occurred in 11 patients, which were used for the sensitivity analyses. The authors found that CK‐MB and hs‐cTnT predict PMI with a comparable diagnostic accuracy and discriminatory power >95%. They also attempted to propose an improved, more sensitive threshold of hs‐cTnT for PMI (120× the upper reference limit of normal [URL] instead of 10 × URL). Of note, they rightfully state this proposal might be inappropriate, primarily due to the low PMI event rate. Nevertheless, the authors should be commended for their thorough analysis with standardized postoperative echocardiography and laboratory measurements at 1, 6, 12, 24, and 48 h after surgery. Still, in our opinion, some remarks and nuances are appropriate.

Although the authors clearly state that the inclusion of a variety of cardiac surgical procedures is a strength of the study, it might also be perceived as a limitation. The UDMI‐4 and a more in‐depth report by the *European Joint Working Groups on Cardiovascular Surgery and the Cellular Biology of the Heart*,^8^ actually exclusively formulated criteria for PMI diagnosis after isolated CABG. Although the authors of the consensus statements self‐admittedly chose cTn cut‐off concentrations more or less arbitrarily, the scientific base for PMI diagnosis after non‐CABG procedures is even weaker. In the study by Niclauss et al., the incorporation of mitral valve surgery, where bicaval cannulation and atriotomy inherently result in myocardial damage, and septal myectomy, where the goal of the procedure is to induce myocardial damage, seem methodologically worrisome. Thereby, even in isolated CABG itself, important differences in the number of distal grafts (i.e., aortic cross‐clamping time),^9^, ^10^ use of different types of cardioplegia,^11^ and use of cardiopulmonary bypass (i.e., OPCAB) exist.^12^ Especially the use of OPCAB yields significantly lower biomarker concentrations due to the absence of cannulation and cardioplegic arrest, as confirmed in a recent meta‐analyis.^13^ The proposal of a uniform biomarker cut‐off concentrations for the diagnosis of PMI following all types of surgery could therefore even be harmful, as it would potentially underdiagnose OPCAB patients with actual bonafide PMI (as OPCAB results in the lowest expected hs‐cTn release) and misdiagnose mitral valve surgery patients with unjustified PMI (while mitral valve surgery is associated with increased benign biomarker release). This uncertainty holds true for the whole spectrum of cardiac surgical procedures, with these extremes influencing biomarker peak concentrations most.

Another issue is using a gold standard for calibration of the diagnostic modality to identify PMI timely. Historically, many studies have relied on (longer‐term) ECG findings, particularly the development of new pathological Q‐waves on follow‐up ECG, which unfortunately have yielded a relatively low predictive value of prior myocardial infarction.^14^ Other ECG findings as definite ST‐elevation or new conduction abnormalities (left bundle branch block) reflect actual PMI,^8^ but are relatively rare after CABG, and their absence certainly does not rule out PMI. In the current study by Niclauss et al.,^7^ the biomarker dynamics were weighed against the UDMI‐4, which actually incorporates the use of cTn, making the UDMI‐4 an imperfect gold standard for calibration of cTn concentrations. The cut‐off values for cTn proposed by the UDMI (>10 × URL with supporting evidence) and SCAI (>35 × URL with supporting evidence, >70 × URL for isolated rises) were somewhat arbitrarily chosen but mostly relied on studies using long‐term survival as a standard. One of the most important studies providing the foundation for these cut‐offs was a meta‐analysis by Domanski et al.,^15^ where even minor postoperative biomarker increases were predictive of long‐term outcome. This means that the presumption was made that these biomarker increases were related to PMI, and PMI was deemed predictive of long‐term survival. Although long‐term survival is probably the most important clinical outcome, this indirect relation is subjected to potential confounding factors and prohibits the actual comparison to a gold standard.^1^ Thus, the question remains as to which standard biomarker concentrations can be weighed, and a possible answer points in the direction of cardiac imaging. Although several modalities exist, especially cardiac magnetic resonance combined with late gadolinium enhancement accurately predicts infarct size and long‐term outcome.^16^, ^17^ A first attempt in the postoperative phase following CABG was made by Pegg et al.^18^ in a relatively small subgroup analysis, demonstrating the superiority of cTn for detection of periprocedural myocardial necrosis.

Whether we should aspire to use a uniform cut‐off value or even a cut‐off value per procedure, remains unknown for the time being. With the emerging role of *precision medicine*,^19^ an integrated approach to cardiovascular disease, using individuals' characteristics, genetics, and risk factors, it seems intuitive something similar would apply to this important matter of debate. Although only hypothesis‐generating, a patient‐specific model would have the potential to indicate the normal expected range of biomarker increases per patient, prospectively. The base of such a patient‐specific model would exist of type and extent of surgery. As several risk factors, such as sex, age, and renal disease, influence cTn kinetics as well, these would also be amenable for inclusion. Of course, the realization of such a model would be time‐ and cost‐consuming. In addition, its development and calibration would require a significant sample size of patients (especially given the relatively low incidence of PMI) with subsequent external validation, warranting the support of contemporary techniques as artificial intelligence and machine learning.^20^ In addition, an important condition would be that such a prospective patient‐specific cut‐off would be precise and clinically simple to apply. Other potential factors to investigate in the near future, could be the pattern of the cTn release, the steepness of its slope or the absolute or relative changes (delta).^21^ In the meantime, more sensitive general cut‐off concentrations are helpful, for which we depend on groups as Niclauss et al., helping to improve the definition of PMI step‐by‐step.

## CONFLICT OF INTERESTS

Alma Mingels has received nonfinancial support from Abbott Diagnostics and Roche Diagnostics. Other authors declare that there are no conflict of interests.
